# Long-term outcomes of the hip shelf arthroplasty in adolescents and adults with residual hip dysplasia: a systematic review

**DOI:** 10.1080/17453674.2020.1747210

**Published:** 2020-04-02

**Authors:** Koen Willemsen, Christiaan J Doelman, Ali S Y Sam, Peter R Seevinck, Ralph J B Sakkers, Harrie Weinans, Bart C H van Der Wal

**Affiliations:** a Department of Orthopedics, University Medical Center Utrecht, Utrecht;; b Department of Radiology, University Medical Center Utrecht, Utrecht;;; c MRIguidance BV, Utrecht;; d Department of Biomechanical Engineering, Technical University Delft, Delft, The Netherlands

## Abstract

Background and purpose — The shelf arthroplasty was the regular treatment for residual hip dysplasia before it was substituted by the peri-acetabular osteotomy. Yet, evidence regarding the survival of shelf arthroplasty surgery has never been systematically documented. Hence, we investigated the survival time of the shelf procedure until revision to THA in patients with primary hip dysplasia. Factors that influenced survival and complications were also examined, along with the accuracy of correcting radiographic parameters to characterize dysplasia.

Material and methods — The inclusion criteria were studies of human adolescents and adults (> 16 years) with primary or congenital hip dysplasia who were treated with a shelf arthroplasty procedure. Data were extracted concerning patient characteristics, survival time, complications, operative techniques, and accuracy of correcting radiographic parameters.

Results — Our inclusion criteria were applicable to 9 studies. The average postoperative Center-Edge Angle and Acetabular Head Index were mostly within target range, but large variations were common. Kaplan–Meier curves (endpoint: conversion to THA) varied between 37% at 20 years’ follow-up and 72% at 35 years’ follow-up. Clinical failures were commonly associated with pain and radiographic osteoarthritis. Only minor complications were reported with incidences between 17% and 32%.

Interpretation — The shelf arthroplasty is capable of restoring normal radiographic hip parameters and is not associated with major complications. When carefully selected on minimal osteoarthritic changes, hip dysplasia patients with a closed triradiate cartilage may benefit from the shelf procedure with satisfactory survival rates. The importance of the shelf arthroplasty in relation to peri-acetabular osteotomies needs to be further (re)explored.

The concept of shelf arthroplasty as a treatment for hip dysplasia was introduced by Franz König (1891); autologous bone is transplanted extra-articularly to extend the coverage of the femoral head by the acetabulum. Nowadays, shelf arthroplasty that relies on fibrocartilaginous changes of the capsule has mostly been replaced by treatments that reorient the patient’s own hyaline cartilage, the peri-acetabular osteotomy (PAO) being one of the most frequently used treatments (Clohisy et al. 2009). However, evidence proving the superiority of the PAO over shelf arthroplasty is lacking. A systematic review of Clohisy et al. (2009) including 13 studies concerning PAO treatment displayed conversion rates to THA between 0% and 17% during, respectively, an average follow-up of 3 and 11 years. Moreover, the PAO is a relatively invasive procedure that necessitates a long rehabilitation period, requires a long learning curve, and has major complication rates reaching as high as 37% (Clohisy et al. 2007).

A systematic review concerning shelf arthroplasty survival in adolescent and adult patients has never been made. Therefore, the primary objective of this study is to systematically evaluate the long-term survival of shelf arthroplasty in adolescents and adults. As a secondary objective we evaluated factors that influence survival, the amount and type of complications, and the ability to correct radiologically dysplastic parameters to normal levels.

## Material and methods

**Figure 1.**
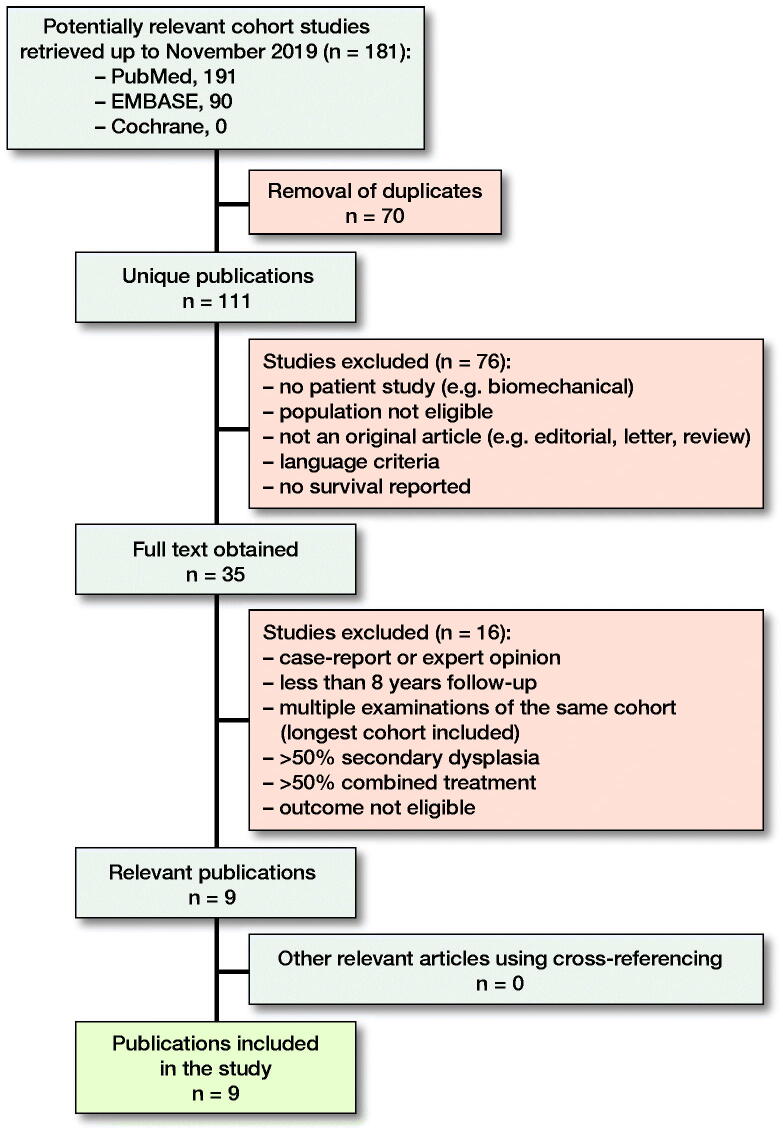
From the 111 unique publications that were found in the systematic literature search, only 9 publications were eligible for this systematic review.

For this systematic review, we consulted the databases Pubmed, Embase, and Cochrane, per search date of November 2019. The term ‘shelf’ was separately combined with the term ‘arthroplasty’ including all known synonyms to minimize the chance of missing articles (see Supplementary data). Obtained articles were imported into a RefWorks database (ProQuest, Ann Arbor, MI, USA). After removal of duplicates the abstracts were read separately by 2 authors (CD, AS) in search of the inclusion criteria (Figure 1).

Inclusion criteria were studies reported in the English language, population human subjects with an average age of 16 years and older with mainly primary (congenital) hip dysplasia, treated with a shelf procedure, and with follow-up of at least 8 years. Studies concerning ≥ 50% secondary hip dysplasia, e.g., due to Down syndrome, Trevor’s disease, Perthes disease, or cerebral palsy were excluded. Studies that used ≥ 50% combined dysplasia treatments, e.g., additional osteotomies, were also excluded because the influence of the combined treatment on the results is not clear. In addition, studies with an average follow-up of less than 8 years, case reports, and reviews were excluded. Studies were excluded only when there was consensus between authors (KW, CD, AS). Finally, cross-referencing was done in the bibliographies of the included studies.

Each published full article was reviewed separately by 3 of the authors (KW, CD, AS). Items reviewed included age, sex, number of patients and hips, study type, level of evidence, type of shelf procedure, type of graft used, amount of patients who were lost to follow-up, combination with other treatments, previous operations, preoperative osteoarthritic state (with scale), failure definition, survival-rates, complications, used surgical indication, amount of conversions to total hip arthroplasty at final follow-up, and the change in hip score (with scale). If documented pre- and postoperatively, the 2 hip parameters (Center Edge Angle = CEA, and Acetabular Head Index = AHI) were also reviewed and displayed graphically. Furthermore, the Newcastle Ottawa Scale (NOS) was used to assess the quality of each study and the average between 2 observers (CD and AS) was documented (Tables 1 and 2).

**Table 1.** Study characteristics

Reference	NOS score	Study design **^a^**	Level of evidence	Type of shelf procedure	Analyzed hips/ patients	Male/ female	Mean age (range)	Combination with other treatment n (%)	Previous operation n (%)	OA scale **^b^**	Preop. advanced OA n (%)
Bartoníček et al. (2012)	8	R	IV	Bosworth (1961)	25/18	1/17	31 (16–52)	0 (0)	2 (8)	TH	2 (8)
Berton et al. (2010)	8	P	III	Modified Roy- Camille (1968)	17/17	NR	34 (20–49)	(100) ^c^	NR	TH	4 (14)
Fawzy et al. (2005)	8	R	IV	NR	76/67	14/53	33 (17–60)	6 (8)	≥ 7	MP	>32 (42)
Hamanishi et al. (1992)	6.5	R	IV	Spitzy (1933)	124/113	12/101	24 (10–53)	33 (27)	8 (7)	NR	NR
Hirose et al. (2011)	7.5	R	IV	Mizuno (1970)	28/26	0/26	34 (17–54)	6 (21)	NR	JOA	0 (0)
Migaud et al. (2004)	7	R	IV	NR	56/48	NR	32 (17–56)	NR	NR	MP	32 (57)
Nishimatsu (2002)	7	R	IV	Spitzy (1933)	119/108	3/105	25 (1–56)	27 (26)	NR	JOA	58 (48)
Saito et al. (1986)	8	R	IV	Mizuno (1970)	27/24	3/21	25 (11–55)	NR	11 (41)	NR	6 (22)
Tanaka et al. (2018)	7	R	IV	Modified Spitzy (1933)	35/32	2/30	31 (19–49)	NR	(0)	TH	0 (0)

NOS = Newcastle Ottawa Scale for assessing study quality; NR = Not reported

**^a^**Study design: P = prospective, R = retrospective

**^b^**OA scales:

JOA = Japanese Orthopedic Association (Takatori et al. 2010) and Oxford Hip Scores (Dawson et al. 1996)

MP = De Mourgues and Patte (1978)

TH = Tönnis and Heinecke (1999)

**^c^**Diagnostic arthroscopy

**Table 3.** Indications for the shelf procedure and negative survival predictors as suggested by the authors

Reference	Surgical indication shelf	Significant negative survival factors
Bartoníček et al. (2012)	Dysplastic centered hip, without osteoarthritic changes, even in patients who are 60 years old	Aspherity, decentration, osteoarthritic changes.
Berton et al. (2010)	Age over 18 years, dysplastic hip, (0° < CE angle < 20°), hip centered with regard to the Shenton line	Osteoarthrosis, CE angle < 0°, subluxation, labral tears (in positive-angle acetabular dysplasia)
Fawzy et al. (2005)	Mild/moderate dysplasia, minimal secondary arthritis	Advanced osteoarthritis, moderate/severe incongruency
Hamanishi et al. (1992)	Age under 30, pre-/early osteoarthritis, stable hip joint,	Age above 30, bilateral dysplasia
	with intact or uninverted labrum	
Hirose et al. (2011)	Moderate dysplasia, without severe osteoarthritis; however, advanced osteoarthritis in combination with femoral valgus osteotomy might be possible	None found
Migaud et al. (2004)	If peri-acetabular osteotomy is not possible because of severe subluxation or incongruency	Severe dysplasia (CE angle < 15°), advanced stage osteoarthrosis
Nishimatsu et al. (2002)	Younger age (however not < 6 years)	Older age, advanced osteoarthritis, height of the shelf
Saito et al. (1986)	Age under 30, no or early degenerative change	Age above 30, severe degenerative changes
Tanaka et al. (2018)	Moderate dysplasia without severe osteoarthritis	Incorrect graft placement (too high)

CE angle = center-edge angle.

Preoperative advanced osteoarthritis was recorded and dichotomized because different scales were used: the Tönnis and Heinecke (1999), De Mourgues and Patte (1978), Japanese Orthopedic Association (Takatori et al. 2010) and Oxford Hip Scores (Dawson et al. 1996). Because of the heterogeneity of this parameter, we distinguished between mild and advanced osteoarthritis. Therefore, on every scale the level that corresponds to advanced osteoarthritis was identified after which the number of patients who were in an advanced state of osteoarthritis were identified (Table 1). Differences in extracted information were discussed between the 3 reviewers and consensus was reached regarding the aspect in question at all times. Authors of included studies were not contacted in the event of missing data.

### Funding and potential conflicts of interest

KW and HW have received research grants from the European Government through the Prosperos project by Interreg VA Flanders—The Netherlands program, CCI grant no. 2014TC16RFCB046 and KW, HW, PS from the Dutch government through the Netherlands Organization for Scientific Research (NWO; Applied and Engineering Sciences research program, project number 15479) in relation to the submitted work. The funders of the study had no role in the study design, data collection, data analysis, data interpretation, or writing of the report. The corresponding author had full access to all the data in the study and had final responsibility for the decision to submit for publication. HW has also received a research grant from the Dutch Arthritis Foundation outside the submitted work. PS has owner shares in MRIguidance BV not related to the submitted work. AS, CD, BW, and RS declare no competing interests. 

## Results

111 unique publications were found in the databases Pubmed, Cochrane, and Embase. 9 studies remained after inclusion and exclusion criteria were applied. Cross-referencing offered no additional articles, resulting in 9 studies analyzed in this study (Tables 1 and 2).

All the studies, except for Berton et al. (2010), are observational retrospective cohort studies without a control group. Berton et al. is a prospective cohort that stratified for the existence of labral tears.

In all studies autologous cortical bone was used and placed superiorly and extra-capsularly to create an extra weight-bearing area and increase joint stability (Nishimatsu et al. 2002, Migaud et al. 2004, Fawzy et al. 2005, Berton et al. 2010, Hirose et al. 2011, Bartoníček et al. 2012, Tanaka et al. 2018). The bone was harvested from the iliac crest (Nishimatsu et al. 2002, Migaud et al. 2004, Bartoníček et al. 2012), the iliac inner (Fawzy et al. 2005) or outer (Hirose et al. 2011, Tanaka et al. 2018) fossa. Unicortical grafts were used by 2 studies (Migaud et al. 2004, Tanaka et al. 2018) and both uni- and bicortical grafts were used by 1 study (Fawzy et al. 2005). A tectoplasty was performed in 2 studies by raising a vertical flap and filling the space with cancellous bone (Nishimatsu et al. 2002, Hirose et al. 2011). Cancellous bone was packed above the shelf by 3 studies (Fawzy et al. 2005, Bartoníček et al. 2012, Tanaka et al. 2018). Migaud et al. (2004) contained the cortical shelf by securing it with a small bent plate. The operation time of 55 minutes (35–75) was only documented by Bartoníček et al. (2012). Some studies combined the shelf arthroplasty in a minor part of their total population with a varus or valgus osteotomy of the proximal femur (8–27%) (Hamanishi et al. 1992, Nishimatsu et al. 2002, Hirose et al. 2011). Berton et al. (2010) combined the shelf procedure with diagnostic arthroscopy solely to image the labral condition. No surgical alterations were made.

Preoperative indications varied widely (Table 3). Early arthritis secondary to dysplasia was used as indication in 3 studies (Hamanishi et al. 1992, Nishimatsu et al. 2002, Hirose et al. 2011). Pain was used as a preoperative indication by Fawzy et al. (2005) and Bartoníček et al. (2012). Radiographic parameters were used for preoperative indications by 4 studies (Migaud et al. 2004, Berton et al. 2010, Bartoníček et al. 2012, Tanaka et al. 2018); the diagnosis ‘congenital dislocation and subluxation of the hip’ was used by 1 study (Saito et al. 1986).

**Figure 2.**
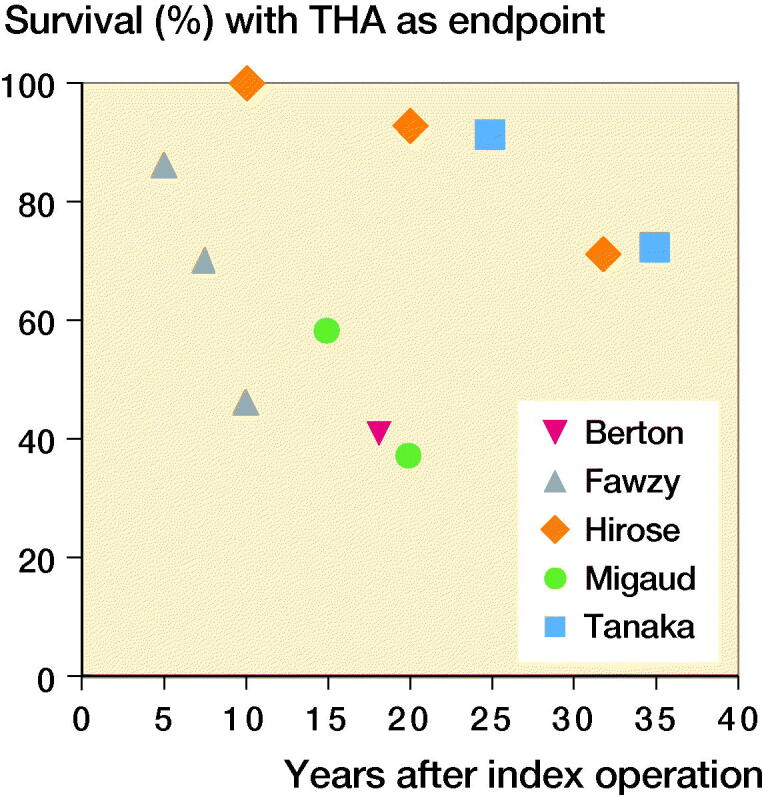
Survival of shelf arthroplasties with years to THA as endpoint. Data for these Kaplan–Meier survival analysis results were extracted from the articles.

Kaplan–Meier survival analysis with THA as endpoint (Figure 2) was documented by 5 studies (Migaud et al. 2004, Fawzy et al. 2005, Berton et al. 2010, Hirose et al. 2011, Tanaka et al. 2018). Fawzy et al. (2005) analyzed 76 hips from 67 patients with an average age of 33. From those shelf procedures, 86% lasted 5 years, 70% lasted 7.5 years, and 46% lasted 10 years until revision to THA. However, many hips showed advanced narrowing of the joint space preoperatively with 32 hips graded as grade IV on the De Mourgues and Patte scale (1978) (> 50% joint space narrowing). When the 44 hips with preoperative grade 3 or less only were analyzed, they found a substantially higher survival percentage of 97% at 5 years and 75% at 10 years.

Berton et al. (2010) used a prospective trial to investigate the effect of the CE angle and labral tears on the shelf arthroplasty survival in a small group of patients. From the 18 patients with an average age of 34 years, 8 hips were converted to a total hip replacement at 18 years’ follow-up. This was significantly higher in the group with labral tears with 7 hips (85%) converted in 18 years of follow-up, as compared with the group without labral tears with 1 hip (17%) converted in 18 years of follow-up.

Migaud et al. (2004) analyzed 56 hips in 48 patients with an average age of 32 at the time of shelf arthroplasty. From their hips, 58% survived 15 years, and 37% managed to survive for 20 years. Similarly to Fawzy et al. (2005), Migaud et al. (2004) treated 32 hips at baseline with grade III or higher on the De Mourgues and Patte scale (1978). These 32 severely osteoarthritic hips had a significant lower survival than the 24 lower graded hips, respectively 27% and 83% survival at 18 years.

Hirose et al. (2011) analyzed 28 hips in 26 patients with an average age of 34 years. All had some amount of osteoarthritis but not one was graded as severe. 29 patients (51%) were lost to follow-up and were therefore not included in the analysis. All hips lasted to the 10-year mark, 93% lasted 20 years, and 71% lasted until 32 years’ follow-up. Hirose et al. (2011) undertook additional survival analysis for clinical evaluation and stage of joint space narrowing of 28 hips. The survival with joint space narrowing < stage 3 on the (0–4) scale of the JOA as an endpoint was 79% at 10 years, 54% at 20 years, and 21% at 32 years. Survival with a pain score of 20 (scale 0–40) as an endpoint was 100% at 10 years, 86% at 20 years, and 51% at 32 years.

**Figure 3.**
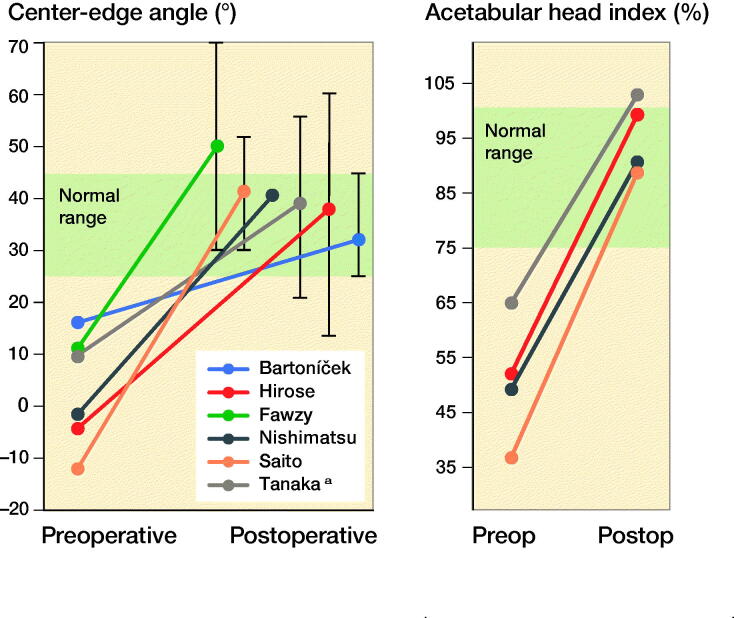
The left panel) displays the average center-edge (CE) angle and the right panel the acetabular head index (AHI) pre(operative) and post(operative). Whiskers display postoperative center-edge angle ranges in relation to the healthy normal/target zone (green areas). ^a^ No range reported, 2 SD was taken as alternative.

Tanaka et al. (2018) analyzed 35 hips in 32 patients with an average age of 31 years and no cases of advanced osteoarthritis at the time of shelf arthroplasty. The hip survival with conversion to THA as the endpoint was 91% at 25 years and 72% at 35 years. The survival with a Tönnis osteoarthritis score of 3 or higher as the endpoint was 74% at 25 years’ follow-up.

All articles reported the number of conversions to THA but only in regard to their average follow-up. This ranged from 2% conversions in 10 years to 47% conversions in 16 years (Table 2). Fawzy et al. (2005) and Migaud et al. (2004) stratified their outcomes for the grade of preoperative osteoarthritis and Saito et al. (1986) for severe degenerative changes. All found a negative effect of preoperative advanced osteoarthritis on the outcome of the shelf arthroplasty.

In general, functional outcomes between studies were difficult to compare because of heterogeneous clinical scoring methods and patient characteristics (Table 2). Moreover, evaluation time points in relation to the surgery or the number of patients per evaluation were often not reported. The average functional outcome improved postoperatively (Saito et al. 1986, Hamanishi et al. 1992, Hirose et al. 2011, Bartoníček et al. 2012, Tanaka et al. 2018) and this improvement lasted up to the final follow-up (Saito et al. 1986, Hamanishi et al. 1992, Hirose et al. 2011) even after 25 years of follow-up (Tanaka et al. 2018).

Most studies documented radiological angles. Perioperative CE angles were documented in all studies and the AHI was measured in 5 studies (Saito et al. 1986, Nishimatsu et al. 2002, Berton et al. 2010, Hirose et al. 2011, Tanaka et al. 2018). All studies that documented both preoperative and postoperative values found a postoperative increase in average CE angle and/or AHI (Figure 3). However, the range of surgical correction achieved was not always within the target values (Figure 3). Both radiographic parameters and functional outcomes were documented in 4 manuscripts (Nishimatsu et al. 2002, Hirose et al. 2011, Bartoníček et al. 2012, Tanaka et al. 2018), yet no relation between radiographic scores and function was reported.

Rehabilitation and postoperative weightbearing was documented in 6 studies with no clear consensus between the different studies (Saito et al. 1986, Hamanishi et al. 1992, Fawzy et al. 2005, Hirose et al. 2011, Bartoníček et al. 2012, Tanaka et al. 2018). Non-weightbearing walking started at 2 days to 6 weeks, partial weightbearing started at 6 to 8 weeks and full weightbearing started at 10 weeks to 6 months.

The complication rate and the background information on the complications were reported by 4 articles. No major complications were encountered (Table 4).

**Table 4.** Reported complications of shelf procedure

Reference	n (%)	Complications
Bartoníček et al. (2012)	5 (20)	Paresthesia lateral femoral cutaneous nerve (disappeared over time)
	2 (8)	Too large a graft (limited external rotation of 1 hip) Partial resorption of graft (still sufficient coverage)
	1 (4)	Extra screw fixation Non-displacement fracture of graft (after a fall)
Fawzy et al. (2005)	10 (13)	Meralgia paraesthetica
	4 (5)	Nonunion and graft breakage
	3 (4)	Superficial wound infection
	2 (3)	Bursa over metalwork (femoral osteotomy)
	1 (1)	Wound hematoma, knee stiffness after traction, flexion contracture, deep venous thrombosis, heterotopic ossification, pulmonary edema
Migaud et al. (2004)	5 (9)	Non-unions
	2 (4)	Temporary peroneal palsies
	2 (4)	Sacroiliac pain
Saito et al. (1986)	2 (7)	Fracture of the base of the reflected outer cortex of the ilium
	2 (7)	Wrong shelf placement

## Discussion

The aim of this systematic review of the shelf arthroplasty was to describe long-term survival, the ability to correct hip dysplasia radiologically, complications, and surgical indications used. The shelf arthroplasty is considered a simple procedure with a THA-free survival of up to 72% over a 35-year period, provided the right surgical indication is used.

The THA-free survival of the shelf procedure reported in this review is comparable to those of the PAO while not being associated with major complications (Clohisy et al. 2009). However, different approaches of the PAO such as the adductor-sparing approaches could result in better recovery of the patient and fewer complications, yet long-term follow-up is still sparse (Murphy and Millis 1999). When evaluating the 5 out of 9 articles that undertook a Kaplan–Meier analysis as part of their survival analysis, the shelf procedure shows surprisingly high survival results (Figure 2). Especially so when noting that both Migaude et al. (2004) and Fawzy et al. (2005) had a high number of patients with severe preoperative osteoarthritis and Berton et al. (2010) had many cases with an existing labral tear. Both the advanced osteoarthritic and labral tear patients had significantly inferior results as compared with patients without osteoarthritis or labral tears. When fewer patients with advanced osteoarthritis were included, as in the studies of Hirose et al. (2011) and Tanaka et al. (2018), the THA-free survival percentage even reached 72% at 35 years of follow- up. These survival results are in line with a recent study by Holm et al. (2017), who reported very long shelf survival rates in children and adolescents. That study was not included in this systematic analysis because the average age of 56 patients (70 hips) was only 12 years (5–22), an average age that was too low for the inclusion criteria. Holm et al. (2017) reported a THA-free survival percentage of 100% at 20 years, 83% at 30 years, and up to 22% at 50 years. In a separate report from the same hospital, Terjesen (2018) made a sub-analysis for the age group > 12 years (average age 16.1 years). The Kaplan–Meier analysis showed a survival of 100% at 20 years, 72% at 30 years, and 32% at 40 years of follow-up. However, because it concerned a sub-analysis many specifics were not given (e.g., number of patients, sex, average follow-up, combinations with other treatment, previous operations, preoperative osteoarthritis scale, clinical hip score, and lost-to-follow up) and therefore the study was not included in this review. The shelf survival values resemble or are even better than PAO survival in the long term (Schramm et al. 2003, Hasegawa et al. 2014, Lerch et al. 2017). Nonetheless, the shelf arthroplasty is considered a salvage procedure, while the peri-acetabular osteotomy is considered to be joint-preserving surgery. Once again, this raised the question as to whether the shelf procedure should be reconsidered in the palette of treatment options for residual hip dysplasia.

Klaue et al. (1993) noticed that a normal CE angle on a radiograph after a shelf arthroplasty is commonly an overestimation when compared with the true femoral coverage on a CT scan. Therefore, parameters such as the CE angle and the AHI might be overestimated. Nevertheless, new 3D planning and evaluation techniques can overcome difficulties in graft placement and improve the effectiveness of correcting the radiological dysplastic parameters in all dimensions (Figure 3). However, it should be noted that the shelf arthroplasty does not change the hyaline cartilage but rather induces fibrocartilaginous metaplasia of the joint capsule to increase the amount of weight-bearing tissue.

Evaluation of the literature shows substantial limitations. First, the level of evidence was low: 8 out of 9 articles were retrospective with level IV evidence and only Berton et al. (2010) was prospective with level III evidence (Table 1). Low-level evidence is common in orthopedics studies as different surgical techniques are often difficult to compare (Obremskey et al. 2005). The included studies used 6 different modifications of the shelf procedure and all had a different postoperative rehabilitation process. The effects of these differences on the outcome were not clear. Second, the investigated population could be considered a limitation as 5 out of 9 studies were completed in Japan, which has a population with a well-known higher incidence of hip dysplasia (Nakamura et al. 1989). Furthermore, far more women participated in the studies investigated, which could have influenced the results, but none of the included studies stratified for sex.

Another limitation could be the search syntax. Additional unknown nomenclature for the shelf arthroplasty could have influenced the effectiveness of the search syntax. However, cross-referencing did not provide any additional articles, causing the impact of this aspect to be low, presumably.

Lost to follow-up was not documented in Fawzy et al. (2005) and Nishimatsu et al. (2002). Therefore, selection bias could have occurred. Only 2 studies documented the number of patients who died before final follow-up. Berton et al. (2010) reported 2 “unrelated” deaths and Migaud et al. (2004) noted 2 deaths without further explanation.

Another type of selection bias may arise from the lack of consensus on the correct indication for performing a shelf procedure. For example, studies that included patients with incongruency and advanced osteoarthritis showed lower survival of the shelf arthroplasty (Migaud et al. 2004, Fawzy et al. 2005). Saito et al. (1986), Berton et al. (2010) and Bartoníček et al. (2012) included only a few patients with severe osteoarthritis (8–22%), Nishimatsu et al. (2002), Migaud et al. (2004) and Fawzy et al. (2005) included roughly half of their patients with severe osteoarthritis (42–57%), while Hirose et al. (2011) and Tanaka et al. (2018) included no patients with severe osteoarthritis. Differences were also found in inclusion of aspheric hips (Migaud et al. 2004) or spheric hips (Bartoníček et al. 2012), younger patients (Saito et al. 1986, Hamanishi et al. 1992, Nishimatsu et al. 2002) or older patients (Berton et al. 2010) even up to their 6th decade (Bartoníček et al. 2012). An additional evident selection bias was introduced by Migaud et al. (2004) who considered shelf arthroplasty as salvage only in patients not eligible for a peri-acetabular osteotomy.

## Conclusion

The shelf arthroplasty is competent in restoring radiographic hip parameters to normal levels, increases functional outcomes, and is not associated with major complications. When selected on minimal osteoarthritic changes, adolescent and adult hip dysplasia patients may benefit from the shelf procedure with satisfactory survival rates. Therefore, based on the findings in this review, the indications for shelf arthroplasty should more often be considered in the treatment of residual hip dysplasia, especially with regard to the difficult-to-perform peri-acetabular osteotomy surgery. Given the constant development of 3D-planning techniques, shelf placement can even be further optimized and therefore may increase its clinical effectiveness.

## Supplementary Material

Supplemental Material

## Figures and Tables

**Table:** Table 2. Study characteristics

First author	Study country	Analyzed hips/patients	Years follow-up mean (range)	Conversions to THA during follow-up, n (%)	Clinical outcome scale ^a^	Hip score pre- (range)	Hip score post- (range)	Final score (years)	Lost to follow-up n (%)
Bartoníček	Czech Republic	25/18	15 (10–23)	4 (16)	HHS	68 (56–82)	90 (76–100)	NR	NR
Berton	France	17/17	16 (16–18)	8 (47)	PMA	NR	NR	NR	1/18 (6)
Fawzy	England	76/67	11 (6–14)	22 (30)	OHS	NR	NR	NR	NR
Hamanishi	Japan	124/113	10 (5–25)	2 (2)	JOA	73	NR	86 (10)	NR
Hirose	Japan	28/26	25 (20–32)	5 (18)	JOA	76	NR	92 (5) > 80 (20)	29/57 (51)
Migaud	France	56/48	17 (15–30)	25 (45)	PMA	NR	NR	NR	5/53 (9)
Nishimatsu	Japan	119/108	24 (15–41)	11 (9)	JOA	NR	80	68 (NR)	NR
Saito	Japan	27/24	13 (5–19)	2 (7) **^b^**	PMA	13	16	15 (18)	7/31 (23)
Tanaka	Japan	35/32	26 (16–36)	10 (28)	JOA	82	> 90	86 (25)	NR

NR = Not reported

**^a^** Outcome scales:

HHS = Harris Hip Score (Harris 1969)

JOA = Japanese Orthopaedic Association (Tanaka 1978, Takeda et al. 2006)

PMA = The Postel–Merle d’Aubigné (Merle d’Aubigné 1990)

OHS = Oxford Hip Score (Dawson et al. 1996)

b Additional undefined surgery

## Supplementary data

Search strategies are available as supplementary data in the online version of this article, http://dx.doi.org/10.1080/ 17453674.2020.1747210
