# Supplementary material for: Long-term outcomes of the hip shelf arthroplasty in adolescents and adults with residual hip dysplasia: a systematic review
Source: Acta Orthop. 2020 Apr 2;91(4):383–9. doi: 10.1080/17453674.2020.1747210 (PMC8023942; doi:10.1080/17453674.2020.1747210)
Supplement: Supplemental Material [file IORT_A_1747210_SM4462.pdf]

Supplementary data

Supplementary data

PUBMED	*Search terms*
# 1 =	shelf*[Title/Abstract] AND
# 2 =	(((((((((arthroplast*[Title/Abstract]) OR procedure[Title/Abstract]) OR surgery[Title/Abstract]) OR operation[Title/Abstract]) OR acetabuloplast*[Title/Abstract]) OR hip[Title/Abstract]) OR osteotom*[Title/Abstract]))
# 3	#1 AND #2
# 4	(slotted acetabular augmentation[Title/Abstract]))
# 5	#3 OR #4
# 6 =	((((Hip[Title/Abstract]) OR Coxa[Title/Abstract]) OR Hips[Title/Abstract]))
# 7 =	(dysplas*[Title/Abstract])
# 8 =	# 5 AND # 6 AND # 7

EMBASE	*Search terms*
# 1 =	shelf*:ab,ti
# 2 =	(arthroplast*:ab,ti OR procedure:ab,ti OR surgery:ab,ti OR operation:ab,ti OR acetabuloplast*:ab,ti OR hip:ab,ti OR osteotom*:ab,ti)
# 3	#1 AND #2
# 4	(slotted:ab,ti AND acetabular:ab,ti AND augmentation:ab,ti)
# 5	#3 OR #4
# 6 =	(hip:ab,ti OR coxa:ab,ti OR hips:ab,ti)
# 7 =	dysplas*:ab,ti
# 8 =	# 6 AND # 7 AND # 8

COCHRANE	*Search terms*
# 1 =	''shelf*'':ti,ab,kw
# 2 =	(''arthroplast*'':ti,ab,kw OR ''procedure'': ti,ab,kw OR ''surgery'': ti,ab,kw OR ''operation'': ti,ab,kw OR ''acetabuloplast*'':ti,ab,kw OR ''hip'': ti,ab,kw OR ''osteotom*'':ti,ab,kw)
# 3	#1 AND #2
# 4	(''slotted'': ti,ab,kw AND ''acetabular'': ti,ab,kw AND ''augmentation'': ti,ab,kw)
# 5	#3 OR #4
# 6 =	(''hip'': ti,ab,kw OR ''coxa'': ti,ab,kw OR ''hips'': ti,ab,kw)
# 7 =	''dysplas*'':ti,ab,kw
# 8 =	# 6 AND # 7 AND # 8
